# Barriers and Facilitators to Walking and Physical Activity Among American Indian Elders

**Published:** 2011-04-15

**Authors:** Craig N. Sawchuk, Joan E. Russo, Andy Bogart, Steve Charles, Jack Goldberg, Peter Roy-Byrne, Dedra Buckwald, Ralph Forquera

**Affiliations:** Dean Health System; University of Washington, Seattle, Washington; University of Washington, Seattle, Washington; University of Washington, Seattle, Washington; University of Washington, Seattle, Washington; University of Washington, Seattle, Washington; University of Washington, Seattle, Washington; Seattle Indian Health Board, Seattle, Washington

## Abstract

**Introduction:**

Physical inactivity is common among older American Indians. Several barriers impede the establishment and maintenance of routine exercise. We examined personal and built-environment barriers and facilitators to walking and physical activity and their relationship with health-related quality of life in American Indian elders.

**Methods:**

We used descriptive statistics to report barriers and facilitators to walking and physical activity among a sample of 75 American Indians aged 50 to 74 years. Pearson correlation coefficients were used to examine the relationship between health-related quality of life and barriers to walking and physical activity after adjusting for caloric expenditure and total frequency of all exercise activities.

**Results:**

Lack of willpower was the most commonly reported barrier. Elders were more likely to report personal as opposed to built-environment reasons for physical inactivity. Better health and being closer to interesting places were common walking facilitators. Health-related quality of life was inversely related to physical activity barriers, and poor mental health quality of life was more strongly associated with total barriers than poor physical health.

**Conclusion:**

We identified a variety of barriers and facilitators that may influence walking and physical activity among American Indian elders. More research is needed to determine if interventions to reduce barriers and promote facilitators can lead to objective, functional health outcomes.

## Introduction

Physical inactivity is high among certain US populations, especially those who are older (1), members of racial/ethnic minority groups (2), and economically disadvantaged (3). Physical activity is a modifiable health behavior that can reduce chronic disease risk (4). Personal, social, and environmental barriers may partially account for discrepancies in physical activity rates among certain populations (5).

Population-based studies note that 33% of American Indians report not engaging in any leisure-time physical activity, compared with 28% of all other racial/ethnic groups (6). Objective measures of physical activity indicate that most American Indian adults do not meet the national benchmarks for recommended physical activity levels, and further reductions in physical activity are associated with advancing age and increasing body mass index (7). Previous investigations of American Indians have found that older age, poor social support, unsafe neighborhoods, lack of access to places to exercise, and competing caregiver responsibilities are also associated with lower activity levels (2,8-11). These studies do not generalize well to older American Indians because they investigated younger (11) and female cohorts (8-11), with small sample sizes (2,9). Older American Indians are at an even greater risk of physical inactivity than younger cohorts (12) and may face unique challenges in establishing healthy routines. Furthermore, many of these studies did not investigate factors that may be associated with promoting increased engagement in leisure-time physical activity.

Enhancing our understanding of population-specific barriers and facilitators is a key step toward improving health promotion efforts, especially given that American Indians have rates of cardiovascular disease (17%) and diabetes (20%) that are nearly 2 to 4 times higher than those of whites and other racial/ethnic groups (13). In this study, we examined self-reported barriers and facilitators to walking and physical activity among American Indian elders. We also assessed the relationship between health-related quality of life and barriers to physical activity.

## Methods

### Study design

American Indian elders completed a poststudy survey approximately 9 months following their initial enrollment in a 6-week randomized physical activity trial (14). Surveys were mailed out and returned between January and August 2006. The survey included updated demographic information, the Short Form 12 of the Medical Outcomes Survey, Community Healthy Activities Model Program for Seniors (CHAMPS) Questionnaire, Barriers to Being Physically Active Quiz, information on current walking behaviors, and additional questions on barriers and facilitators to walking and physical activity. Participants who returned the completed survey were compensated with a $20 grocery gift card.

### Participants

A total of 125 American Indian elders aged 50 to 74 years who participated in the randomized trial were mailed the poststudy survey. All study recruitment efforts and procedures were conducted at the Seattle Indian Health Board (SIHB), a large urban primary care facility for American Indians and Alaska Natives in Seattle, Washington. Inclusion criteria for the study included age (50-74 y), sedentary lifestyle (responding no to the question, "Have you been physically active for the past 6 months?"), ability to walk without assistance, lack of medical contraindications to walking, and living within a 2-hour driving radius of the SIHB. We obtained approvals for the randomized trial and poststudy survey from the institutional review board at the University of Washington and from the SIHB privacy board.

### Measures

**Demographic characteristics**

Self-reported demographic data on age, sex, marital status, education level, and current smoking status were collected at the time of the poststudy survey. A research study coordinator assessed participant height and weight at the end of the randomized trial and calculated body mass index (BMI) from height and weight.

**Health-related quality of life**

The Short Form 12 of the Medical Outcomes Survey (SF-12) is a 12-item measure of health-related quality of life during the past 4 weeks (15). The Physical (PCS; α = .81) and Mental Health (MCS; α = .85) Component Summary scores were used in this study. The parent measure of the SF-12, the SF-36, has been previously validated with other studies pertaining to American Indians (16).

**Walking and physical activity**

We included 4 items on current walking behavior that assessed weekly frequency, personal reasons for walking, places to go walking, and likelihood of walking alone. Another 4 items assessed personal and built-environment barriers and facilitators to walking. Respondents were encouraged to report as many barriers and facilitators as applicable.

The CHAMPS Questionnaire is a 41-item measure assessing light, moderate, and vigorous physical activities (17). Respondents reported their weekly participation in activities for the previous 4 weeks, yielding 4 summary scores: total caloric expenditure for all exercise activities, total caloric expenditure for moderate-intensity exercise activities, frequency of all exercise activities, and frequency of moderate-intensity exercise activities. Although CHAMPS had been well-validated with older and other racial/ethnic minority populations, it had not been previously validated with American Indians.

The Barriers to Being Physically Active Quiz (18) is a 21-item measure assessing the following barriers to physical activity: 1) lack of time, 2) social influence, 3) lack of energy, 4) lack of willpower, 5) fear of injury, 6) lack of skill, and 7) lack of resources (eg, recreational facilities, exercise equipment). Each domain contains 3 items, with a total score range of 0 to 63. Respondents rate the degree of activity interference on a 4-point scale, ranging from 0 = “very unlikely” to 3 = “very likely.” Internal consistency for the total score was .87 in this sample. Subscale α coefficients ranged from .77 for “lack of skill” to .45 for “lack of resources.” The source of the lower α coefficient for the lack of resources subscale was the following item: “If we had exercise facilities and showers at work, then I would be more likely to exercise.” More than 75% of the elders were currently not working, so most viewed this item as nonapplicable. A total score of 5 or more on any subscale was considered an important barrier (18). The Barriers Quiz has not been previously investigated with American Indians.

### Statistical analyses

We compared the responses of participants who completed the poststudy survey to those who did not complete the survey in terms of demographic, health, and psychological variables. Descriptive statistics were generated for the entire sample on all study variables and for each sex separately. The responses of male and female respondents were compared on variables relating to demographics, health status, SF-12 components, walking behaviors, personal activity barriers and facilitators, and built-environment activity barriers and facilitators. Chi-square analyses and *t* tests were used for categorical and continuous variables, respectively. We examined the association between the SF-12 component scores and the barrier quiz by using Pearson correlation coefficients, the CHAMPS total caloric expenditure, and total frequency for all exercise activities subscales as covariates. A *P* value of <.05 indicated statistical significance. Analyses were conducted by using SPSS 18.0 (SPSS, Inc, Chicago, Illinois).

## Results

### Participant characteristics

Seventy-five of the 125 elders completed the survey (60%). We found no significant differences on any demographic, health status, or physical activity variables between participants who completed the survey and those who did not. Men and women were similar on all variables, except BMI and asthma frequency, which were significantly higher among women than among men (Table 1).

### Self-reported walking behaviors

Seventy-three percent of respondents reported walking each week; the average walking frequency was 3.4 days (standard deviation [SD], 2.7) per week. Approximately 40% reported being able to walk a quarter of a mile without assistance. More than two-thirds of respondents walked in their neighborhoods to get to and from places; 64% walked to the grocery store, 55% walked for exercise or recreation, and approximately 30% walked to visit others. More than half of the elders (53%) reported that they usually walked alone. Elders were more likely to report walking on sidewalks in both commercial (53%) and residential (52%) areas compared with walking on road shoulders in commercial (10%) and residential (18%) areas.

Most elders reported walking for exercise on a weekly basis, reinforcing findings that walking is a common form of physical activity among this age group (19). Among personal barriers to walking, lack of energy was reported nearly twice as often as any other barrier. Built-environment barriers were infrequently reported by the elders, and they showed little differential among the assessed built-environment barriers.

### Self-reported barriers and facilitators to walking and physical activity

Elders reported an average of 1.0 of 9 personal barriers to walking (SD, 1.0); lack of energy, no one to walk with, bad weather, and lack of interest in walking were the most frequently reported barriers (Table 2). Approximately one-third of the sample reported no personal barriers to walking. Elders also reported an average of 0.7 of 7 built-environment barriers to walking (SD, 1.0); dangerous street crossings, too many hills, and no interesting places to walk were the most commonly reported barriers. Fifty-seven percent reported no built-environment barriers to walking.

Elders reported an average of 2.3 personal facilitators to walking (SD, 1.5). Better health, pleasant weather, someone to walk with, and more energy were the most frequently reported facilitators. Fifteen percent reported no personal facilitators. Elders also reported an average of 2.3 built-environment facilitators to walking (SD, 1.3), and only 5% of the sample reported no built-environment walking facilitators (Table 3). The most common facilitators were being closer to interesting places, shops, and parks.

We found no significant sex differences on either the total scale score or the total number of important barriers. Therefore, both sexes were collapsed into a single sample for all subsequent analyses. The lack-of-willpower subscale had the highest mean, representing the largest self-reported barrier, followed by the lack-of-resources and the social influence subscales (Table 4). Likewise, lack of willpower (47%), social influence (36%), and lack of resources (33%) subscales were reported as being the most important barriers (scores >5) in this sample.

The associations of the SF-12 component scores with the Barriers to Being Physically Active Quiz and current walking behaviors were assessed by using the CHAMPS total caloric expenditure and frequency of all exercise activities as covariates (Table 5). The PCS was negatively correlated with fear of injury and lack of energy. Poor physical health on the PCS was also associated with difficulties walking one-fourth mile and a greater number of built-environment barriers to walking. The MCS was also inversely correlated with the full-scale score on the Barriers to Being Physically Active Quiz and 5 barrier domains, suggesting that poor mental health was associated with lack of willpower, lack of energy, social influence, lack of time, and lack of resources. Finally, the total number of self-reported personal barriers to physical activity was associated with lower MCS scores.

## Discussion

Correlational analyses revealed unique but conceptually logical associations between activity levels and barriers with the PCS and MCS. Even after controlling for self-reported physical activity, significant associations emerged between specific barrier domains and the PCS and MCS. Poor physical health was most strongly related to various physical outcomes and built-environment barriers, whereas poor mental health was significantly associated with social or motivational outcomes and personal barriers to walking. Further, poor mental health quality of life also yielded a much stronger association with overall barriers than did poor physical health. Social support (8,20) and emotional functioning (21) tend to be positively associated with higher rates of physical activity. Problem-solving efforts that address building social support and finding walking partners may be particularly important for older, sedentary people.

Consistent with previous studies (2), personal facilitators to walking largely involved health and social reasons. Both physical and aesthetic features of the built environment are consistently associated with physical activity and walking (22-24). American Indian elders also frequently endorsed many of these built-environment facilitators to walking. Furthermore, they regarded the availability of benches and places to rest as an important environmental feature, a finding that could be helpful in planning built environments that can assist older and medically compromised populations in pacing their physical activity.

Lack of recreational resources was also reported by elders as a significant barrier. Previous studies have noted that racial/ethnic minority and economically disadvantaged communities are 3 to 4.5 times less likely to have available recreational and exercise facilities compared with predominately white and higher-income communities (25). Basic physical features of the neighborhood environment, such as sidewalks, lighting, green space, and aesthetics, are associated with higher levels of physical activity (23,24,26), and community interventions that modify the built environment to promote physical activity can lead to objective increases in walking (27). Indeed, respondents in our study reported a 5-fold increase in self-reported walking preference when sidewalks were present in both residential and commercial areas. The integration of objective measures of both the built environment and physical activity levels is an area for future research. Advances in global positioning technology offer a potential tool for further inquiry (28).

On the Barriers to Being Physically Active Quiz, lack of willpower emerged as the most highly and frequently reported subscale, whereas fear of injury was the lowest-rated subscale. This contrast underscores the importance of using motivational enhancement strategies to bolster physical activity levels in medically ill populations. Previous studies have also found that low motivation is a commonly voiced reason for physical inactivity among older, racial/ethnic minority populations (2,19). In an adult sample of American Indian women, those reporting higher levels of self-efficacy in their ability to be physically active were approximately 2 to 3 times more likely to meet physical activity requirements outlined by the Centers for Disease Control and Prevention and the American College of Sports Medicine (11). Further, another independent sample of American Indian elders similarly noted that low motivation and low self-esteem were common reasons for not walking (2). Interventions to promote exercise that incorporate motivational enhancement are effective in increasing physical activity levels among sedentary adults, and these principles can be easily integrated into primary care (29). The finding that people with higher self-efficacy perceive fewer personal, social, and environmental barriers to physical activity underscores the importance of enhancing self-efficacy in at-risk populations, as this may help to compensate for objective barriers to physical activity (26). Future research should address the efficacy of motivational enhancement and confidence-building interventions to assist elders in reaching and maintaining nationally recognized physical activity guidelines.

Our study has several limitations. First, we assessed urban-dwelling American Indian elders, so our findings may not generalize to other American Indian populations. Second, we conducted within-group, cross-sectional analyses that limit statements regarding the directionality of the relationships. It remains unclear if self-reported barriers and facilitators to walking are stable over time, or if physical activity interventions can produce desirable changes in these outcomes. Third, we did not collect neighborhood data that could have provided a more objective measure of built-environment features that either promote or inhibit physical activity. Fourth, although the data were collected 9 months after enrollment in the randomized activity trial, participation in a study specifically designed to increase walking behavior could have influenced our current findings. Assessing these domains in a new community sample of American Indians might address this uncertainty. Fifth, CHAMPS and the Barriers Quiz have not been previously validated with older American Indians, and therefore questions arise about the cultural relevance of these measures. Finally, many of the built-environment items assessed were not frequently reported by the elders, so we may have missed other barriers.

Despite these limitations, this study contributes to the emerging literature on barriers and facilitators to walking in an at-risk and historically understudied population. Assessing barriers and facilitators to walking and physical activity is an important, early step toward improving health outcomes in older populations. To our knowledge, this is the first study to examine the Barriers to Being Physically Active Quiz among American Indian elders, a population known to be at risk for sedentary lifestyles and metabolic disorders. Primary care interventions that incorporate exercise prescriptions, use motivational enhancements, and address individual-specific barriers to physical activity offer promise in reducing disease risk in sedentary people. Randomized trials can specifically assess personal and built-environment barriers to walking and whether these barriers can be reduced in a clinically meaningful manner through coaching on physical activity and problem solving.

## Figures and Tables

**Table 1:** Characteristics of American Indian Elders, by Sex, Seattle Indian Health Board, 2006.

Characteristic	No. (%), n = 75	Women, No. (%), n = 58	Men, No. (%), n = 17	Test Statistic^a^
**Demographics**				
**Age, y, mean (SD)**	58.5 (5.7)	58.9 (5.8)	57.1 (5.4)	1.15
**Married or cohabitating **	17 (22.7)	15 (25.9)	2 (11.8)	0.85
**At least some college **	47 (62.7)	36 (62.1)	11 (64.7)	0
**Employed full or part time **	17 (22.7)	13 (22.4)	4 (23.5)	0
**Annual household income <$5,000 **	23 (30.7)	17 (29.3)	6 (35.3)	0.16
**Health status**				
**BMI, mean (SD)**	31 (6.3)	31.4 (6.7)	28.5 (4.0)	2.23 (.05)^b^
**Current smoker **	24 (32.0)	18 (31.0)	6 (35.3)	0.04
**Self-reported chronic diseases**				
Osteoarthritis	42 (56.0)	35 (60.3)	7 (41.2)	1.26
Diabetes	21 (28.0)	17 (29.3)	4 (23.5)	0.03
Hypertension	31 (41.3)	24 (41.4)	7 (41.2)	0.00
High cholesterol	19 (25.3)	16 (27.6)	3 (17.6)	0.49
Asthma	16 (21.3)	16 (27.6)	0	4.54 (.05)^b^
Heart disease or stroke	11 (14.7)	8 (13.8)	3 (17.6)	0.00
Cancer	9 (12.0)	8 (13.8)	1 (5.9)	0.21
At least 1 of the 7 above conditions	61 (81.3)	48 (82.8)	13 (76.5)	0.05
At least 2 of the 7 above conditions	46 (61.3)	37 (63.8)	9 (52.9)	0.28
**SF-12 component scores**				
Physical (PCS), mean (SD)	40.3 (11.2)	39.4 (11.4)	43.1 (10.7)	1.18
Mental health (MCS), mean (SD)	43.6 (12.0)	43.0 (12.0)	45.7 (12.1)	0.82

Abbreviations: SD, standard deviation; BMI, body mass index; SF-12, the Short Form 12 of the Medical Outcomes Survey; PCS, Physical Component Summary Score; MCS, Mental Health Component Summary Score.

a For frequency measures, χ^2 ^test was used; for straight comparisons, *t* tests were used.

a This value was significant at *P* < .05.

**Table 2:** Personal and Built-Environment Barriers to Walking for American Indian Elders (N = 75), Seattle Indian Health Board, 2006

**Barrier**	No. Reporting Barrier (%)
**Personal **	
Lack of energy	22 (29)
No one to walk with	12 (16)
Bad weather	12 (16)
Lack of interest in walking	11 (15)
Lack of time	10 (13)
Having to carry heavy items	5 (7)
Child care responsibility	2 (3)
Unattended dogs	1 (1)
Need car after work	0 (0)
**Built-environment **	
Dangerous street crossing conditions	9 (12)
Too many hills	9 (12)
No interesting places to walk	9 (12)
No safe places to walk nearby	7 (9)
Too much traffic	6 (8)
Distances are too great	5 (7)
No sidewalks	4 (5)

**Table 3:** Personal and Built-Environment Facilitators to Walking for American Indian Elders (N = 75), Seattle Indian Health Board, 2006

**Facilitator**	No. Reporting Facilitator (%)
**Personal **	
Better health	36 (48)
Pleasant weather	26 (35)
Someone to walk with	24 (32)
More energy	24 (32)
More interest in walking	18 (24)
Not owning a car	11 (15)
More knowledge about benefits of walking	11 (15)
A dog to walk with	11 (15)
Carrying only light items	11 (15)
More time	7 (9)
No or less childcare responsibility	3 (4)
**Built-environment **	
Closer to interesting places	31 (41)
Closer to shopping places	26 (35)
Closer to parks	25 (33)
Benches and places to rest	23 (31)
Good lighting at night	17 (23)
Closer to walking trails	15 (20)
Interesting architecture to look at	8 (11)
Longer crosswalk signals	8 (11)
More trees along streets	8 (6)

**Table 4:** Barriers to Being Physically Active Quiz^a^ for American Indian Elders (N = 75), Seattle Indian Health Board, 2006

Barrier	Mean (SD)
Lack of willpower	4.4 (2.5)
Lack of resources	3.6 (2.4)
Social influence	3.5 (2.5)
Lack of energy	3.2 (2.6)
Lack of time	2.7 (2.4)
Lack of skill	2.6 (2.5)
Fear of injury	2.2 (1.9)
Total score	22.1 (11.7)
**No. of important barriers^b^**	2.0 (1.9)

Abbreviation: SD, standard deviation.

a Higher scores indicate more barriers; scale scores range from 0 to 9 for each subscale and 0 to 63 for the total score.

b Range of scores is 0 to 7; higher scores indicate more barriers. A score of ≥5 on subscales indicates an important barrier. This result relates to the average number of subscales that individual elders reported as being a significant barrier (2.0 subscales). It will vary from elder to elder.

**Table 5:** Pearson Correlations Between SF-12 Component Scores, Barriers to Being Physically Active Quiz, and Current Walking Behaviors, for American Indian Elders (N = 75), Seattle Indian Health Board, 2006^a^

**Measure**	Pearson Correlation	

	SF-12 Physical Component Score,^b^ *r* (*P *value)	SF-12 Mental Health Component Score,^b^ *r* (*P *value)
**Barriers to being physically active quiz^c^**		
Lack of willpower	−0.06 (.60)	−0.50 (<.001)
Lack of resources	0.02 (.89)	−0.24 (.04)
Social influence	−0.14 (23)	−0.30 (.01)
Lack of energy	−0.24 (.04)	−0.37 (.001)
Lack of time	0.01 (.95)	−0.23 (<.05)
Lack of skill	−0.18 (.12)	−0.10 (.38)
Fear of injury	−0.47 (<.001)	−0.21 (.07)
Total score	−0.22 (<.05)	−0.40 (<001)
**Current walking behaviors**		
Any walking during usual week	0.05 (.66)	0.02 (.86)
No. of days walking per week	0.001 (.97)	0.03 (.78)
Difficulties walking 0.25 mi	−0.62 (.001)	−0.10 (.42)
Walking alone	0.14 (.23)	0.04 (.75)
Personal barriers to walking	0.01 (.92)	−0.36 (.002)
Built-environment barriers to walking	−0.22 (.06)	−0.09 (.44)

Abbreviation: SF-12, Short Form 12 of the Medical Outcomes Survey.

a Adjusted for the total caloric expenditure and frequency of all exercise activities in the Community Healthy Activities Model Program for seniors.

b Higher scores on the SF-12 indicate greater self-rated quality of life.

c Higher scores on the Barriers Quiz indicate greater self-reported barriers to physical activity.
